# Talking morphine: Pain and prognosis in Indian cancer care

**DOI:** 10.1111/maq.12895

**Published:** 2024-10-20

**Authors:** Nickolas Surawy‐Stepney

**Affiliations:** ^1^ Department of Global Health and Social Medicine King's College London London UK

**Keywords:** cancer, India, morphine, pain, palliative care

## Abstract

Pain can be a pervasive feature of cancer, particularly in regions such as India, where the disease is rarely detected in its early stages. Yet over recent decades, morphine, a “gold standard” pain medicine, has been rarely used in India. This article draws on anthropological discussions of clinical disclosure in Indian cancer care to complicate assertions that this is because pain is missed or ignored by healthcare workers. Instead, in a context where the disclosing of prognoses is partial and indirect, I argue that morphine has gained a communicative function. Typically withheld until the “end of life”, the drug has come to be read as a death sentence. It has become an analgesic *and* a prognosis. It is an object that talks in situations where direct communication is often avoided.

“*As clinicians we were reluctant to prescribe morphine, especially during the disease process. We used to think morphine is for end‐of‐life care*.”

“*More educated people, that speck of thought immediately comes “my patient has been put on morphine, it means he or she may be terminally ill”… if I can avoid putting [a patient] on morphine I will, [as] they at times just put it back*…”

*Senior medical oncologist in a government cancer center*

## INTRODUCTION

Pain can be a pervasive feature of cancer, particularly in regions where the healthcare infrastructure is not set up to detect the disease in its early stages (Banerjee 2020; Caduff & Van Hollen, 2019). Compounding this problem, areas that exhibit high rates of untreated advanced cancer are often also those in which the availability of strong analgesics is low (Livingston, 2012). India is a prime example. While Indian healthcare can be as technologically advanced as anywhere in the world, hospitals that provide strong pain medicines can be difficult to reach for much of the population, particularly those living in rural areas. This is especially striking in the case of morphine, a drug effective for cancer pain that is very cheap to manufacture and distribute (Ruiz‐Garcia & Lopez‐Briz, 2008). A widely cited Human Rights Watch report (2009) estimated that only around 4% of cancer patients in India requiring the drug could obtain it.

India's punitive Narcotic Drugs and Psychotropic Substances (NDPS) Act is a key legislative reason for this material scarcity. Enacted in 1985, amid national and international concern about rates of drug addiction (Deshpande 2009), it erected significant regulatory hurdles for hospitals wishing to legally stock the drug (Khosla et al., 2012). Fearing the heavy punishments mandated for failing to maintain any of the multiple licenses required to do so, many healthcare practitioners subsequently ceased procuring it (Rajagopal et al., 2001). Manufacturers likewise moved away from a product so stringently controlled, refining the vast majority of manufactured morphine to its downstream products such as codeine. In 2020, the country produced almost 13 tons of morphine, yet only around 1.7% was used in palliative care (International Narcotics Control Board, 2021).

In an attempt to mitigate these impacts, the NDPS Act was amended in 2014, yet in a 2018 study of 1600 cancer patients attending major centers across the country, Katherine Doyle and colleagues (2018) found pain to still be “inadequately treated” in 67% of cases. Subsequent to this amendment, public health studies have commonly suggested that the drug remains rarely used in part because the pain exhibited by patients is either dismissed by healthcare workers (Lebaron et al., 2014) or is otherwise inadequately assessed (Dureja et al., 2017).

Drawing on 10 months of ethnographic fieldwork based around a North Indian cancer hospital between 2019 and 2022, this article provides an additional analysis to supplement and complicate these suggestions. Opposing the assertion that the attention given to pain is inadequate, I argue that careful pain assessment is crucial to much of the work done within cancer centers. Pain was present throughout the clinical encounters I witnessed, it was both acknowledged and scrupulously drawn into diagnostic work. This observation thus demands a further question. If pain is not ignored, what explains the relative reluctance to prescribe—and accept—a strong analgesic like morphine? Why, as indicated in the second quote heading this paper, would a doctor avoid prescribing morphine for fear of it being simply “put back” (typically returned to a hospital) by a patient or a member of their family?

Building upon anthropological discussions of disclosure in Indian cancer care, I argue that in this context morphine assumes a role beyond that of just an analgesic. Where cancer is often untreated until its advanced stages, where the disease and its treatment carry high financial, emotional, and social burdens, and where public cancer hospitals creak under the weight of patient numbers, the disclosure of accurate clinical information is a fraught undertaking. Focused largely on cancer diagnosis, scholars have displayed how cancer's revelation is not a normative act of truth‐telling; it can reshape social relationships in sometimes catastrophic ways (Banerjee, 2020). Such communication is characterized by avoidance and fear, on both the side of the doctor (Van Hollen, 2018) and that of the patient (Bright, 2015). Here, I suggest that prognoses can be understood in the same way; prognostic disclosure is a speech act characterized by hesitance, silence, and partial revelation, often mediated through kin.

This seeds fertile ground for other objects or actions to function in the communicative register. Drawing on E. Summerson Carr's (2011) analysis of the semiotics of clinical relations, I suggest that within this context of ambiguity, doctors manage their clinical work to maintain a notion of possibility, by pursuing courses of “curative” treatment such as chemotherapy, alongside moderate pain relief such as tramadol or buprenorphine. In so doing, they signal the value of repeated returns to the hospital for continued treatment. Yet, as the first of the two ethnographic quotes suggests, morphine has been typically reserved for the “end of life”, withheld until treatment aimed at curing the disease has failed and other forms of analgesia have been halted. While many medical objects and practices carry meaning in a situation where direct prognostic communication is fragmentary (a chemotherapy or radiotherapy regimen carries the promise of recovery, for example), morphine has come to convey what neither doctor nor patient wishes to express: a death sentence. In this way, morphine carries a predictive force; it is an analgesic *and* a prognosis, “talking” when direct communication is otherwise avoided.

This argument has three parts. By demonstrating a typical morning in the hospital's outpatient department (OPD), I provide an alternate reading to the implication made in public health literature that morphine remains rarely used because the pain exhibited by Indian patients is either missed by healthcare workers or is otherwise ignored. I suggest instead that pain is implicated in the formation of diagnoses and managed in particular ways. Second, I explore the ambiguities that surround the disclosure of diagnoses and prognoses in these clinical spaces, and how this opens a space for alternate, indirect modes of communication. Finally, by examining the stepwise approach to pain management employed in the hospital and the particular semiotics of care, I show how morphine, withheld until the “late stages” of disease, thus threatens to articulate what otherwise often goes unsaid.

## MORPHINE IN A GOVERNMENT HOSPITAL

The hospital that served as the primary field site for this research sits in a small city in Northern India. The largely rural population that it treats is, in comparison with that of other North‐Indian states, relatively well served by local, public primary healthcare. Larger tertiary centers such as this, however, are more sporadically spaced and fairly small. The cancer center in which I worked was the largest of its kind in the state, yet it had only 50 beds. This is relatively few for a center that sees roughly 2500 patients per year, most of whom are treated as outpatients in the various clinics that work through long patient lists each morning. The doctors had the perception that most of their patients were poor or middle‐class, noting that those with the means usually went straight to one of the larger hospitals or to private clinics in the bigger cities of the region. The communicative functions of morphine that I analyze relate mostly to those patients the doctors regarded as more “educated”, yet the effects permeate the care provided to all attendees.

This particular government center started as a tumor clinic in the late 1970s, and it acquired its first radiotherapy equipment roughly a decade later. The hospital was assigned the status of “Regional Cancer Centre” in the early 2000s, and after 2 years, it gained the authority to train doctors in the postgraduate qualification of “MD in Radiotherapy and Oncology.” As a government cancer center, it is one of the few in the state to have been granted a license to stock strong opioid drugs such as morphine. The stocks held by the hospital were stored separately from other drugs, away from the pharmacy, which had ready supplies of “weaker” analgesics such as buprenorphine and tramadol. Of other opioids considered “strong”, synthetic drugs such as fentanyl are more commonly found in private healthcare facilities, largely because their high relative price renders them attractive within settings determined by profit. In this hospital, however, as in many state cancer hospitals, morphine was the mainstay of “strong” opioid pain management. Despite the presence of a newly created palliative care department, it was the oncologists of the cancer center that most often prescribed, distributed, or withheld the drug. In part because of its novelty, few patients were transferred from the oncology lists to that of the lone palliative care doctor. In his words, “only the patients the oncologists don't know what to do with” came to his attention.

While more senior physicians were generous in their time and expertise, the bulk of their clinical duties involved overseeing complex clinical procedures such as radiotherapy, brachytherapy, and chemotherapy. These radiotherapy and oncology trainees, termed “resident doctors”, were therefore my primary interlocutors. They were the people who carried out much of the relevant labor within the hospital; running the outpatient clinics, conducting ward rounds, maintaining a log of morphine prescriptions, and dispensing the drug to patients when it had been prescribed.

## PAIN IN THE OUTPATIENT DEPARMENTS

The new patients’ OPD was the last in a line of three small rooms that served the vast majority of the center's patients who did not occupy the two wards. The first two of these offered their services to returning male and female patients respectively, and the final to all new patients who came to the hospital. After registration, this room was where patients first interacted with the doctors and had their bodies examined and their symptoms discussed. The information collected in these encounters entered the brown hospital folders that followed each patient and formed the basis on which many future decisions were made ‐ clinical referrals, treatment plans, admittance, and discharge.

Each morning, this room bustled with activity. Such was the volume of people hoping to have their time with the doctors that a line formed filling the corridor back 10 or 15 metres. The door was almost always open, the queue of patients spilling in. To deal with this quantity of potential patients, two or three of the resident doctors worked around the small desk at any one time. Each copying notes from patients’ prior healthcare records into the files of the cancer center, taking careful histories, conducting physical examinations—sometimes ushering others out of the room when these involved more intimate areas of the body, sometimes conducting them on a bed behind a small green curtain that cordoned off one corner.

When a doctor began their work, they picked up a new file from those piled on the desk. A shouted name alerted those outside as to who was now being considered. The patient to whom this work‐related entered only when their medical history and examination needed to be done. More often, the call brought in one of the patient's attendants with extra information such as a scan, or the results of a test carried out at one of the many private diagnostic facilities dotted around the state's larger towns. This was a protocol not immediately apparent to many of those waiting in the corridor; on several occasions frail‐looking patients were helped through the doorway only to be repeatedly waved out and one of their attendants gestured in instead.

As the first patient of the day was examined, a second resident called in another, the OPD establishing itself at a pace. A steady queue of people stood in the open door. With two doctors in the room, history‐taking and examinations took place at double speed. Questions were fired from each side of the desk, their answers scribbled onto sheets of paper.

“Blessed with four daughters”^1^, one of the residents read from the notes written by the referring doctor of a female patient waiting in the corridor. This could have been written either at a peripheral hospital or in the main public hospital, from where many patients are sent. It was noticeable that a family history in its fullest sense was taken from each patient. This was not a history merely of diseases in the family, but a deeper examination of the structure and solidity of these ties. How many children does this person have? Do they still live at home, how old are they? Is the patient married, and if so, what is the occupation of their spouse? The relatives to which the questions refer were often in the room, and they helped with the responses.

These questions are used to not only think through potential pathologies (those directed at women about their number of children, the status of their marriage, and so forth are explained as relating to risk factors for cervical cancer, a major form of the disease in the state) but also to help each patient navigate the diagnostic and therapeutic landscape. The case of a young boy with suspected lymphoma illustrates this. As the second resident silently copied the notes from a previous hospital into the new file, he asked the boy's mother about fever and night sweats, and the job of the boy's father, who was not present. “Does he have a government job?” he asked. This was directly related to the price of diagnostics and the choices that would have to be made in this regard. A PET scan in a private facility costs Rs. 18,000 [roughly $255 at the time of fieldwork], the doctor added.

As soon as this interaction was complete, another patient entered and perched on the red stool beside the desk. The doctors asked about pain. Notes were written. In this instance, pain was the first symptom discussed. As many of the doctors explained, pain is likely what drives most people to come to the hospital. The concerned doctor pointed to his side and asked about that location. He talked to the second resident about renal cell carcinoma, querying pain, hesitance, and frequency of urinating. “Dard kee davaee?” He asked the patient. Any pain medications? The history continued: appetite, cigarette smoking, cancer in the family? Hold out your hands, the doctor instructed, and the patient obliged. He looked them up and down and turned them over, muttering inaudibly before reaching for a stethoscope and listening to the sounds at the base of her lungs.

After the doctor washed his hands, the notes were then finished and placed within the file. This is the moment when pain first appears as an inscription; the words that note the site and severity of that pain, the paper on which they are written, and the folder in which the paper is kept forming what Shagufta Bhangu (2019, 161) terms “habitats” of pain. It also marks a multiplying of pain; in its recording, it has expanded from a phenomenon of day‐to‐day personal life (one that is often interruptive if not debilitating) to additionally become a series of carefully noted physiological signs.

Within medical anthropology, recent studies that have addressed the question of pain in Indian medical settings have largely been situated in clinics or specialties that take this aspect of medicine as their primary focus—either in cancer palliative care (Banerjee, 2020) or in nascent specialties focusing on chronic pain (Bhangu, 2019). In this work, scholars have drawn out the multiple modalities of pain, including its psychological, social, pathophysiological, familial, and material registers. Pain and palliative care doctors, they show, carefully work with these multifactorial aetiologies to address this aspect of the disease. As I suggest here, though, many people who engage with cancer services in this setting do not find themselves under the care of the few pain or palliative care doctors working in the region. Instead, much of this work is done within cancer centers themselves.

By contrast, public health literature, the strand of scholarship that has taken on the question of pain and analgesic use in this setting most often, commonly proposes that the material scarcity of morphine and other strong opioid pharmaceuticals is explainable through pain's affective absence. Authors suggest that the cancer pain exhibited by Indian patients is either overlooked by healthcare workers or is otherwise disregarded. Virginia LeBaron et al. (2014, 518), for example, in a study conducted with oncology nurses, suggest that “often during fieldwork patients were observed suffering in pain, but participants did not seem to ‘see’ the same reality; obvious pain… was dismissed as not a problem, or attributed to psychological causes”. Spurred by the low levels of opioid pain relief distributed in the country, Gur Prasad Dureja, of the Delhi Pain Management Centre, conducted a review into the pharmacological pain management of patients in India. “In cancer patients”, he writes, “inadequate pain assessment was considered a major barrier (20%–80%) …The physicians either do not use an appropriate method to measure the pain intensity or fail to appropriately classify the pain” (Dureja et al., 2017, 179). In both examples, there is no equivocation on the primacy of a (particularly biological) pain in these settings, but both suggest that the pain experienced by patients is somehow not “reaching” those tasked with providing care. In this view pain either goes “unseen” or “unmeasured”.

In demonstrating these clinical interactions in quick succession, I aim to do two things. On the one hand, I give a sense of the constant work that these morning OPD sessions entail; the work done in these scenarios is hurried, its participants frequently dealing both with rapid patient consultations as well as queries from other doctors, hospital staff and visitors who call questions through the open door. But I also provide an alternate reading to public health claims about the affective absence of pain. As we have seen, pain questions are incorporated within general histories, and the information gained no doubt goes beyond that which is easily recorded by the visiting ethnographer. The patients who come to these clinics carry with them information from referring doctors that can be treated with skepticism within the cancer center. The doctors working at this stage of the hospital process are therefore very thorough in ascertaining information that might help clarify the diagnosis and stage of the disease.

Medical science has developed supposedly universal tools for the quantification of pain (the McGill pain questionnaire, for example, see Melzack (1975)), but in the context of the structural limitations manifest in this small room—its limited space, overburdened staff and surfeit of need—such tools are woefully inefficient. Instead, doctors explain that they rely on the means displayed above: simple scoring scales and manual physical examinations. Dwaipayan Banerjee (2020, 95) writes of similar practices at the All India Institute of Medical Sciences (AIIMS) in Delhi, one of the country's premier medical institutions. He describes the ways such formalized, “scientific” modes of knowing as moderated pain scales can miss the “tacit knowledges” (Polanyi, 1967) conveyed in physical, tactile encounters. Fingers pressing flesh elicit winces or sharp breaths. Despite the lack of obvious quantification, one should not underestimate the substantive information gathered from consultations such as these. The means of information transfer, while not immediately open to quantification and audit, rests on the skill and experience of the doctor.

The problem of pain's knowability, the crux of the wider debates within the humanities scholarship on the subject (Elaine Scarry's (1985) work on the individual and inexpressible nature of pain as well as subsequent anthropological analyses that conversely demonstrate its social allocation (Das, 1993) and experience (Livingston, 2012)), is not reflected in the clinical practices observable in the cancer hospital. In this context, pain is a key indicator. Pain questions are dotted throughout the clinical histories being taken, their function perhaps not to ascertain distress but to aid in diagnosis. Doctors note both whether the patient confirms or denies pain and how they react to the touch and tactile pressure of the physical exam, to help erect the diagnostic scaffolding.

In this manner, the doctors interact with physiological pain in a way that reflects the function of the cancer center. Bhangu (2019), in her study of the Institute for Pain Management in Kolkata, argues that the constant twinning of paper records and physical body (as is observable in the cancer hospital) allows multiple diagnoses to be made. In this way, multiple assessments and knowledge of pain emerge. The doctors in the cancer center OPDs however, display a different goal from their colleagues in pain management: they seek a unitary diagnosis for which a singular treatment plan can be formulated. While the physical locations of pain are also sought in the cancer center, this is done with the specific intention of ascertaining the nature, stage, and location of any underlying pathology. It is this pathology that will then often be the recipient of therapeutic attention.

## CLINICAL DISCLOSURE

Almost all those who attend the OPDs do so with the help of others, often family or friends, who provide the majority of non‐medical care in and around the hospital. But as already indicated, these attendants are also drawn into the processes of diagnosis and treatment. The case of an elderly man who had come with his son demonstrates this. Arriving with one of the doctors, he took his place at the congested desk, finding a corner to balance the new folder collected moments earlier from the records office. The history began immediately, with the resident asking about pain, its location, duration, and severity (using a scale of zero to one hundred). “Have your parents had cancer? What about your children?” the doctor queried. As his general and symptomatic history was being recorded, the man paused to cover his face with a flannel and coughed for a minute or so. The questions nevertheless persisted, his son stepping in to answer.

The doctor continued with some questions about employment. Dark liver spots above this man's eyebrows stood out beneath his white hair. He removed his shoes and was helped to stand and move behind the green screen. As he was examined, his son peered over the barrier. With the examination complete, the doctor washed his hands, and the patient struggled back into his shoes to be helped out of the door. The notes were then finished and placed within the file.

In the corridors and consulting rooms of the cancer hospital, I had seen on the forms and notes that new patients brought with them the various logos of diagnostic services and other small private healthcare providers that they had consulted. I asked one of the residents about the relationship between the cancer center and these private services. “There are not many large private hospitals around here”, he replied, “but a few smaller ones. These places are only interested in curative treatment. They are run for profit. Often,” he continued, “they won't tell people their diagnosis. They will hint only. ‘You may have cancer so you should go to the cancer hospital.’ It is us who must do the dirty work.”

Whether conducted privately or in public facilities, these scans often end up on the doctors’ desks in the new patients’ OPDs of more specialized cancer hospitals, carried by people for whom a diagnosis is uncertain, indicated obliquely through instructions about follow‐up treatment or further diagnostics. Having looked at the CT scans of the man he had examined, one of the residents made it clear to me that this was indeed a case of quite advanced cancer. Likely “stage 3b”, on a scale that runs from one to four.

The doctor called the son back into the room, asking him to close the door behind him. He explained that his father had cancer. Stage 3b. The son replied with a quiet *“tik hai sir* [OK]”. The doctor outlined the potential treatments, chemotherapy, and radiotherapy, and the son greeted each with the same whispered affirmation. He took the pile of notes and scans and put them in his bag. His father subsequently joined the men in the male OPD.

Patients often do not know the details of their possible conditions. Primary and secondary physicians, particularly in the private sector, do not always pass on their suspicions of cancer. In the cancer center too, the transmission of this information is not straightforward. It was not the elderly man with pain in his chest who was told that it was being caused by “stage 3b” cancer. That information was relayed to his son; the elderly man remained waiting in the corridor. Other people were being examined in the OPD as this was explained; thus, the closing of the door only partly functioned to maintain some form of privacy. More significantly, it shielded this man from being the direct recipient of this disclosure. Likewise, while it was explained to me that the numerical indicator “3b” signified severe disease on a possible range of 1–4, this explicit positioning was not made clear to the patient's son. Nor indeed was any direct prognosis offered. Instead, potential therapeutic approaches were set out.

This form of indirect communication has become a focus of the growing palliative care movement within the country, informed by ideas around patient empowerment in clinical decision‐making. “Doctors in India often say, ‘families won't be able to take all of the information, so we just tell them bits,’” said an advocate at a palliative care conference held at the cancer center in late 2019, in an impassioned appeal to delegates. “But we have to learn to say that ‘your patient is dying.’ This is often what the family is thinking anyway. Proper communication is needed. [But] many doctors say ‘Oh I don't have time. I have 150 patients in my OPD.’” A doctor working in a palliative care organization in Delhi similarly remarked that part of their work is ensuring that the people they treat know the details of their disease and prognosis. In part, this was framed as a cultural difference. “*In our culture, perhaps not in yours, people hide diagnosis and prognosis from their patients*”, he reflected in an interview. “*The doctors usually tell the family members. And that too, in a blunt way… But they are always short of time*.”

The withholding of information around diagnosis and prognosis either in medical settings or between kin is a feature of communication around cancer that is well known in India. As Banerjee (2020); Kristen Bright (2015); Jacob and Matthew (2017); and Cecilia Van Hollen (2018) all show, rather than being simply a question of truth and its obfuscation, concealing cancer is a complex act with many divergent motivations at play. Working in South India, Bright (2015) has displayed how, in a context of scarce financial resources and high treatment costs, the hiding of cancer symptoms can be a form of care: protecting households and wider kinship networks from emotional and economic hardship. Yet these ties constituted here as protective and protective, can also be the source of neglect, violence, and harm. A cancer diagnosis in this manner can function to deepen and intensify painful social relationships (Banerjee, 2019a, 2019b). Likewise, when aetiology is contested and can implicate moral transgression, a cancer diagnosis or poor prognosis can strain not only household relationships but also those between a family and the wider social networks in which it is located (Macdonald, 2015). Any normative evaluation of such concealment is thus complicated. As Van Hollen (2022, 141) demonstrates through her work with lower‐caste women in Tamil Nadu, what comes to be at stake is the “quality of care” that non‐disclosure represents. Certain people, typically those of higher social position, she suggests, experience this concealment as an act of beneficence, a signifier of the protection afforded by both family members and medical professionals. For others, by contrast, the withholding of clinical information can be received as a form of class or caste‐based discrimination, perceived as merely one instantiation of the “impersonal, dismissive and disrespectful attitudes of doctors in public hospitals toward patients who come from predominantly poor, lower‐caste communities” (Ibid: 150).

In the OPD cases related above, we see not only how prognostic communication is mediated through family members, but also how the realities of contemporary hospital practice stymie those very interactions. One can see the constraints in the material conditions of the government cancer hospital, as time‐strapped practitioners work constantly to assess each of the patients queuing for long periods outside the consultation rooms. As Sarah Pinto (2014, 228) notes in her account of madness in North India, clinical conditions produce good diagnosticians, but nevertheless undoubtedly render “partial” the stories that patients may tell, and likewise curtail the practices open to clinicians that might further improve their ability to hear those stories. But it is not just the acquisition of clinical information in the form of medical histories and examinations that is conditioned by such institutional limitations. Communication of diagnoses and prognoses from doctor to patient are also informed by the clinical and social environments in which they take place. In this sense, the frenetic clinical settings in which doctors and patients interact directly shape the knowledge that people hold over the diseases in their own bodies. “Partial” stories of pain, disfigurement and death, hope and recovery, occupy the spaces where there is no diagnostic or prognostic certainty. This space of uncertainty opens the possibility for other means of communication, including through the choices made around analgesia.

## ANALGESIC DECISIONS

For patients, pain is often the reason they travel to the center. Once there, pain is transformed into a series of indicative physiological signs to be carefully noted in the hospital folders. Pain is used as a vital clue to the particularities of underlying disease, for which treatment is then sought. Yet it is also true that strong analgesia is not often prescribed. Instead, the therapeutic routes pursued tend to be chemotherapy or radiotherapy, and strong pain medication is left to be used only when this ostensibly “curative” treatment has ceased. These “curative” stages of treatment extend throughout the disease process; several palliative care specialists in the region whom I interviewed complained that they did not get patients referred to them at early enough stages, some even decrying the fact that chemotherapy or radiotherapy would sometimes be continued right up until the death of a patient.

The primacy of such strategies was described directly by a pharmacist working beside the chemotherapy ward. “Why would we give morphine at the earlier stages of disease?” he asked me. “It won't do anything to help cure it”. There seemed a tacit logic at play here, where pain both resulted from—and was revelatory of—underlying pathology: to treat that pathology was also to therefore address pain. This logic was later made explicit for me in the hospital. The center specializes in radiation oncology, and one morning between procedures, one of the physicists took me aside to explain some of the more easily understood biological mechanisms by which radiation destroys the multiplying cancer cells. “But radiation is also very good for pain”, he added. Elaborating, he told me that radiation damages all human tissue, but healthy tissue recovers while cancerous tissue does not. The implication: treat pathology and you treat pain. This “collapse” of pain into pathology (Livingston, 2012, 120) helps explain the simultaneous acknowledgment of pain and the absence of strong analgesics.

It is not the case, however, that other forms of analgesia are denied to patients. Choices about pain relief are made in highly disciplined ways, carefully following the guidelines set out by the World Health Organization's “analgesic ladder”—a scale formulated in 1986, to improve and standardize pain treatment in cancer care (Ballantyne et al., 2016). It has become the de facto rubric by which analgesics are chosen; reference to it was made in the hospitals, hospices, and palliative care NGOs that I visited in different parts of India. This simple tool recommends a stepwise approach to treating pain, increasing the strength of analgesia only when the drug on a lower rung fails to control it, progressing from drugs such as ibuprofen through “weak” opioids like tramadol or buprenorphine, and only then to “strong” opioids like morphine.

Careful adherence to this international guideline ensured that almost all the patients seen in the OPDs received something on the lower end of the analgesic ladder in their initial stages of treatment. One such example was a lung cancer patient seen in the OPD who had been sent home with buprenorphine patches (step two), tramadol (step two), and paracetamol (step one). “If that doesn't control the pain,” the doctor in charge of his consultation explained, “we'll go up the ladder.”

The hospital drew in patients from across the state, roughly 20,000 square miles of mountainous terrain. As such, should this patient or any like him return home and have their condition deteriorate, they may well not have been able to make the journey back to the hospital to be further assessed. As has been demonstrated by Van Hollen (2022), the possibilities of making repeated journeys to seek healthcare are mediated by gender, class, and caste inequalities. Treatment “default”, to adopt the normative and censorious medical phrase, was common. The higher levels of the analgesic ladder may never be reached not because they are not warranted but because of the structural limitations of an underfunded public health system wherein large and dispersed populations rely on very few advanced tertiary hospitals capable of delivering cancer therapeutics. The distance of these institutions from the homes of many of their patients can make repeat visits highly demanding, thus, rendering the repeat assessment required less likely.

Importantly, this is not ubiquitous across the country. In addition to working primarily in the north of India, I also spent 2 weeks working with a palliative care group in Kerala, where civil society groups, in partnership with the state, organize pain care that can be more aligned with community needs. There, I observed groups of healthcare workers making long daily rounds of the wider community assessing the analgesic requirements of those who had sought out their services. Here, too, the WHO ladder was a constant reference, yet it was used quite differently. Rather than being a scale merely ascended, regular meetings with patients allowed both the ascension from “weak” to “strong” painkillers including morphine, but also the reverse when the pain was deemed “managed”. In mountainous North India, by contrast, the difficulties patients face when accessing healthcare keep a drug like morphine at a firm distance throughout the initial stages of treatment. The WHO ladder functions not only to guide treatment decisions throughout the disease process but also to define the scenarios in which certain drugs are used in regionally specific ways.

That the rungs of the ladder can only be climbed on the occasions when patients are reassessed and their prior analgesia found to be insufficient, combined with a clinical practice “reluctant to prescribe morphine during the disease process”, relegates morphine to advanced stages of disease when any attempt to extend life has ceased. “*Most of the patients we used to put on it* [morphine] *were terminally ill”* explained a senior hospital oncologist in an interview. *“They used to die sooner or later. So now, at times, it has become synonymous with death.”* This reciprocal relationship marks morphine differently from other analgesics. Morphine specifically has become equated solely with the “end of life”. Or, to remove the impersonalizing effect of medical language, death.

## A THERAPEUTIC SEMIOTICS

Describing addiction treatment in the United States, Carr (2011) writes of how therapists and their clients utilize sociomedical scripts to different ends. Healthcare professionals render patient experience amenable to therapeutic intervention by setting linguistic boundaries for its description, while their clients replicate such language to manage their trajectories through an often punitive institutional landscape. In both cases, Carr (2011, 3) notes, clinical work is “in essence, *semiotic* work” (emphasis in original). While modern oncology no doubt engages powerful, life‐altering interventions, the ambiguity around communication that I have described shows that clinical semiotics is a highly important feature of much Indian oncology. As the words of the doctors displayed above demonstrate, they too are well aware of the semiotic functions that their therapeutic practices entail.

Banerjee (2020, 7), in his analysis of the patient experience of cancer in Delhi, suggests that actions such as diagnostic concealment reveal a pervasive orientation towards the subjunctive mood, the “could be”, in the experience of the disease. This move toward realities distinct from lived reality (in which a diagnosis or prognosis may or may not have been made) yet ordered as if possible, provides an ethical mode of action when lived reality, subject to the limitations of social and medical circumstance, is too much to bear. What we see in this instance, I suggest, is a reciprocal set of actions undertaken by medical staff.

To the dissatisfaction of the palliative care physicians, oncologists often pursue extensive regimens of aggressive chemotherapy or radiotherapy until very close to a patient's death. This is no doubt a feature of the interventionist medical training they have received, but it is also in part continued to encourage the repeated engagement of their patients with the hospital. Courses of chemotherapy or radiotherapy are often long, and many doctors fear their patients will not continue for the full duration of their treatment. Yet a further chemotherapy appointment (rather than with a palliative care doctor) nevertheless indicates the maintenance of the *possible*, and so the value in returning to the hospital for yet another round.

Pain is also managed with this intention. To keep the rates of return high, the doctors express a need for results tangible to the people they are treating. “Less pain,” one of the residents explained, “is, for the patient, a sign that the chemotherapy is working. With less pain, they are more likely to come back for the next stage of treatment. This is why we must do pain management simultaneously.” The expectation is that for patients if the pain lingers, they will think that so too does the cancer. The doctors’ treatment of pain is thus partly instrumental—it is not done out of a sense of need; specific (lower rung) analgesia is sometimes prescribed to signal to a patient that the pathology‐focused treatment is working (whether it is or not) and that they should therefore return to continue receiving it.

Through the maintenance of ostensibly “curative” treatment modalities and the management of pain, physicians thus work to establish and maintain a therapeutic (rather than palliative) semiotics, a sense that there may yet be multiple possible outcomes, and that continued engagement with the hospital is therefore necessary. In such a scenario, the extended temporalities of chemotherapeutic or radiotherapeutic regimens postpones the use of morphine. Whether it is in the ambiguity maintained around a diagnosis or prognosis, the maintenance of therapeutic treatment, or the management of pain to convince a patient in the reduction of disease, each is a semiotic gesture towards the continued potential for alleviating cancer, and the value in returning for greater medical care.

To suggest this is not to mistakenly transpose the notion of “hope” and “survivorship” as the dominant imaginaries of cancer from North American and European contexts to that of India. As Banerjee (2020) suggests, where treatment is hard to access at the early stages of the disease, pain is more likely a unifying trope. Yet through acts of concealment, ambiguity, and therapeutic intervention, there nevertheless persists a multitude of possibilities for cancer sufferers and their families. Morphine is thus merely one of many “talking” objects and actions here, but one that can shatter the impression of a possible return to health. When other drugs or procedures hint at rescue, morphine articulates the threat of therapeutic failure, abandonment, and death.

## CONCLUSION

Through careful work with cancer patients, scholars such as Van Hollen (2022) have demonstrated how the most marginalized find ways to assert their agency in the face of both destructive disease and the medical systems established to treat it. Against this perception of unwillingness, delays in (re)visiting hospitals or “incorrect” ways of managing treatment (such as returning pills) are reframed to acknowledge the injustices of a deeply unequal society, of social obligations of caring for others, and the financial constraints of seeking medical attention. In my research, I considered patients—particularly those who received morphine—to be ethically unsuitable participants due to the often highly advanced stages of their disease. As such, here I have focused largely on the perspectives of doctors, analyzing the medical care more broadly offered, and specifically the relative absence of the drug therein.

Scholarship that has sought to understand analgesic practices and the relative material absence of morphine from Indian hospital wards is typified by invocations of an apparent “ignoring” of pain by those working in cancer hospitals. Throughout this article, I have therefore focused on the clinic, showing how pain, even in the biomedical terms of public health, is heavily implicated in clinical work. The pain of the patients who enter the hospital's consulting rooms is not disregarded, nor is it often missed. Instead, it serves a function: to aid in the classification and staging of disease. In the journey from the first encounter with the hospital doctors to the receipt (or not) of medication, the pain of an individual traverses knowledge of several different kinds ‐ qualitative, tactile, as well as quantified and linked to universalized scales such as the WHO analgesic ladder.

This prompts us to consider the temporality not just of illness, but also the therapeutics with which it is met: how a ‘‘symptom’, or “treatment” relates to perceptions of time and “progression”. As I have shown here, pain is acknowledged throughout medical care. In this sense, unlike morphine, pain does not have a clinical temporality specific to the disease process (i.e. limited to “late stage” cancer). It is present throughout, the taking of histories, the staging of disease, and the formulation of treatment plans. Morphine, by contrast, has become temporally bound to the very final stages of life; it has come to indicate the cessation of efforts towards a cure, the renouncing of possibility, and the snuffing out of hope.

In a clinical setting where the circulation of accurate cancer prognostic and diagnostic information between doctor and patient, patient and their attendants, or between kin is often indirect, partial, and hesitant, the social meaning of interventions becomes of vital importance. To prescribe morphine is to cut against the grain of carefully maintained therapeutic semiotics. Intended or not, morphine talks when direct speech is carefully avoided. This communicative function stands against it being widely used.

## ENDNOTE

1 To differentiate quotes taken verbatim from recorded interviews from those recorded in fieldnotes, the former is presented in italics.
